# Comparative efficacy of the front-line anti-HBV drugs in nucleos(t)ide analogue-naive chronic hepatitis B: A protocol for systematic review and network meta-analysis

**DOI:** 10.1097/MD.0000000000020160

**Published:** 2020-05-08

**Authors:** Mao-bing Chen, Hua Wang, Qi-han Zheng, Wei-yan Cui, Hua-lan Xu, Xu-wen Zheng

**Affiliations:** a Department of Emergency; b Department of ICU, Wujin People Hospital Affiliated with Jiangsu University and Wujin Clinical College of Xuzhou Medical University, Changzhou, Jiangsu, PR China.

**Keywords:** chronic, entecavir, hepatitis B, network meta-analysis, nucleos(t)ide analogue-naïve, tenofovir

## Abstract

**Background:**

During the COVID-19 period, there was a huge gap in the understanding of masks between east and west. At the same time, the mechanism of the mask and the effect after use, also appeared differences. The Objective of this Meta-analysis is to systematically evaluate the efficacy of masks for influenza in the community.

**Methods:**

The Web of Science, PubMed, The Cochrane Library, EMBASE and Clinical Trials will be electronically searched to collect randomized controlled trials regarding the efficacy of masks for influenza in the community through Apr 2020. Two researchers independently screened and evaluated the obtained studies and extracted the outcome indexes. Revman 5.3 software will be used for the meta-analysis.

**Results:**

The outbreak is continuing, and we need to be prepared for a long fight. If masks are effective, we need to promote their use as soon as possible. If masks are ineffective, strong evidence should be given. This is an urgent task and our team will finish it as soon as possible.

**Conclusion:**

Provide stronger evidence to solve the problem, should we wear masks or not right now.

## 1 Introduction

Hepatitis B (HB) is an infectious disease caused by hepatitis B virus (HBV) that affects the liver. Chronic hepatitis B (CHB) is the most common form of HB.^[1]^ The clinical manifestations are asthenia, fear of food, nausea, abdominal distension, liver pain, and other symptoms. The liver is large, moderately hard, and tender. Severe cases can be accompanied by symptoms of chronic liver disease, spider nevus, liver palm, and abnormal liver function.^[2,3]^ At least 391 million people, or 5% of the world's population, had chronic HBV infection as of 2017, and more than 1 million patients were first discovered.^[4]^ Over 750,000 people die of CHB each year. About 300,000 of these are due to liver cancer.^[5]^ So CHB is a thorny problem, especially in less developed countries.^[1,6]^

Currently, front-line drugs for CHB include entecavir (ETV) and tenofovir mainly, tenofovir is further divided into tenofovir disoproxil fumarate (TDF) and tenofovir alafenamide fumarate (TAF).^[7,8]^ Suppressing hepatitis B virus is nearly a lifelong undertaking.^[9]^ For the patients without treated (nucleos (t)ide analogue-naive), it is important to choose their first drug that is right for them.^[10]^ In China, the prices of all 3 drugs are close. So in the choice of drugs, we pay more attention to the efficacy and safety of these drugs.^[11–13]^

At present, there is still a lack of relevant systematic research on the efficacy and safety of these 3 drugs for the patients with nucleos(t)ide analogue-naive in the treatment of CHB. It is difficult for patients to choose which drug to take, the newer, the better; or the more expensive, the better? In this study, the efficacy and safety of ETV, TDF, and TAF in nucleos(t)ide analogue-naive CHB patients will be compared to provide a basis for patients to choose the more appropriate anti-viral drug.

## 2 Methods

The purpose of this study is to compare the efficacy and safety of TAF, TDF, and ETV. However, if only pairwise comparison will be conducted, few literatures will be retrieved. Therefore, lamivudine (LAM), adefovir (ADV), and placebo (PLA) will be introduced into this study for comparison, and direct and indirect comparison will make the conclusion more convincing. Certainly, TAF, TDF, and ETV will still be the focus of analysis.

### 2.1 Design and registration

A network meta-analysis will be conducted to evaluate the efficacy of ETV, TDF, and TAF in nucleos(t)ide analogue-naive CHB. This protocol has been registered on the international prospective register of systematic reviews (PROSPERO), registration number is CRD42019143233 (https://www.crd.york.ac.uk/PROSPERO). No ethical approval is required since this study used data that will be already in the public domain.

### 2.2 Study selection

#### 2.2.1 Study type

The study type is randomized controlled trials (RCTs).

#### 2.2.2 Study object

Patients with definite CHB and no prior experience with nucleos(t)ide analogue therapy will be included. The following patients will be excluded: patients who are infected with HIV or other hepatotropic viruses; those who have drug-induced liver diseases, alcoholic liver disease, or autoimmune liver diseases, tumors, serious complications in the heart, kidney, brain, and other organs; and patients who are in pregnant or lactating.

#### 2.2.3 Intervening measure

ETV group: the enrolled patients were given the conventional dose of entecavir 0.5 g/day orally.

TDF group: the enrolled patients were given the conventional dose of TDF 300 mg/ day orally.

TAF group: the enrolled patients were given the conventional dose of TAF 25 mg/day orally.

LAM group: the enrolled patients were given the conventional dose of LAM 100 mg/day orally.

ADV group: the enrolled patients were given the conventional dose of ADV 100 mg/day orally.

PLA group: the enrolled patients were given placebo once daily orally.

#### 2.2.4 Outcome indicator

The following outcomes will be assessed and compared among ETV, TDF, and TAF groups:

(1) normalized ALT,
(2) virological response.

We defined the virological response as the inability to detect HBV-DNA by PCR.

#### 2.2.5 Exclusion criteria

Studies with data that could not be extracted or utilized, studies with animal experiments; and literature reviews were excluded.

### 2.3 Data sources and searches

**Figure 1:**
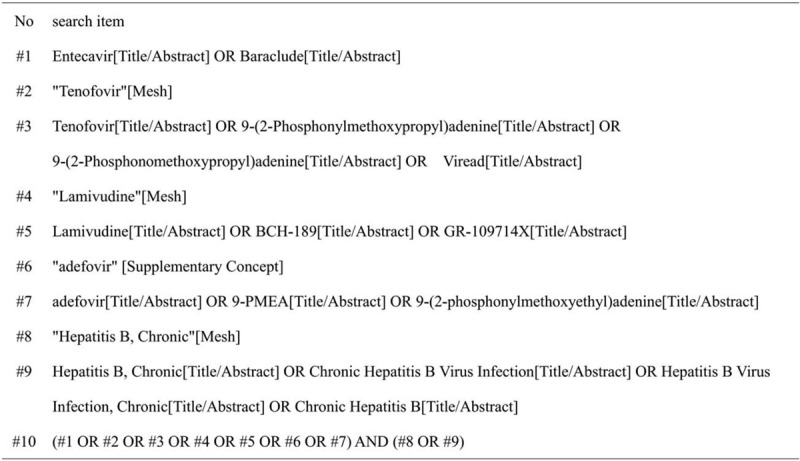
PubMed database retrieval strategy.

We will search English and Chinese language publications through Apr 2020 using the following databases: Web of Science, PubMed, the Cochrane Library, EMBASE, and Clinical Trials. The search terms included “Tenofovir”, “Entecavir”, and “Hepatitis B, Chronic”. In Figure 1, we use the PubMed database as an example.

### 2.4 Study screening, data extraction, and risk assessment of bias

Data will be collected independently by 2 researchers. The unqualified studies will be eliminated, and the qualified ones will be selected after reading the title, abstract, and full text. Then, the research data will be extracted and checked, and disagreements will be discussed or a decision will be made by the authors. The extracted data include the following:

1. basic information of the study, including title, author, and year of publication;
2. characteristics of the included study, consisting of the study duration, the sample size of the test group and the control group, and the intervention measures;
3. the outcome indicators and data;
4. the information needed to assess the risk of bias.

The risk of bias in the included studies will be assessed using the RCT bias risk assessment tool recommended in the Cochrane Handbook for Systematic Reviews of Interventions (5.1.0). This work will also be done independently by 2 researchers.^[14,15]^

### 2.5 Statistical analysis

The bayesian hierarchical model will be used in this study and ADDIS 1.16.8 software will be used for the network meta-analysis. The dichotomous variables will be expressed as the relative risk (RR) as an effect indicator and the estimated value and 95% confidence interval (CI) will be included as effect analysis statistics. The significance level sets at *α* = 0.05. A heterogeneity test will be conducted with the results of each study. If there is no statistical heterogeneity among the results (*I*^2^ ≤ 50%), network meta-analysis will be performed directly. If there is statistical heterogeneity among the results (*I*^2^ > 50%), the source of heterogeneity needs to be found. If we could not find the source of heterogeneity, descriptive analysis will be performed only. Consistency test is needed for network meta-analysis. If *P* > .05, there is no statistically significant difference between direct and indirect comparison, and the results of the 2 are consistent, the consistency model will be used; otherwise, the inconsistency model will be used.^[16,17]^ After the comparison of various interventions, the ranking probability table was used to rank the advantages and disadvantages of the interventions. The STATA 16 software will be used to draw a network diagram of the various interventions, showing direct and indirect comparisons between them. The funnel plot will be drawn to make qualitative judgment of publication deviation.

### 2.6 Subgroup analysis

We will explore whether treatment effects for our primary outcomes are robust in subgroup analyses using the following characteristics: sex, age, race, nationality, duration of medication, etc.

### 2.7 Assessment of publication bias

If more than 15 articles are available for quantitative analysis, we will generate funnel plots to assess publication bias. A symmetrical distribution of funnel plot data indicates that there is no publication bias, otherwise, we will analyze the possible cause and give reasonable interpretation for asymmetric funnel plots.^[18]^

### 2.8 Confidence in cumulative evidence

GRADE system will be used for assessing the quality of our evidence. According to the grading system, the level of evidence will be rated high, moderate, low, and very low.^[19]^

## 3 Discussions

As front-line drugs for the treatment of CHB, the efficacy and safety of ETV, TDF, and TAF should be guaranteed. Nevertheless, for a drug that may require lifetime use, we should give patients more information to help them to make judgments and this can help patients control liver inflammation and inhibit virus reproduction better.

TAF has the same mechanism of action as TDF and is a nucleotide reverse transcriptase inhibitor. Tenofovir bisphosphonates, the active component of tenofovir, inhibit the viral polymerase by directly competing with the natural deoxyribose substrates and terminating DNA strands by inserting DNA. Entecavir is a guanine nucleoside analogue, and its anti-viral pharmacological action is similar to that of tenofovir.^[20]^

For the selection of quantitative analysis outcomes, we pay more attention to the indicators of liver injury and the amount of virus in serum. Because these outcomes are most relevant to functional cure of CHB. For other outcomes, such as HBeAg clearance, HBeAg seroconversion, adverse effects, and so on, descriptive analysis will be conducted.

In the literatures inclusion, if it is a comparison of 2 anti-viral drugs in ETV, TDF, and TAF, we will conduct quantitative analysis, and if it is a comparison of these 3 drugs with placebo, it will still be included in the quantitative analysis. To make the research more credible, we will also conduct quantitative analysis of LAM and ADV in the control group. A placebo control group will be also included in the study. LAM and ADV are common anti-hepatitis B drugs, before these 3 drugs are available. So there are 6 interventions in this study, ETV, TDF, TAF, LAM, ADV, and PLA. Perhaps the placebo group may not exist.

This study will conduct a network meta-analysis of related RCTs, provide evidence on the efficacy and safety of ETV, TDF, and TAF in CHB treatment, and compare the advantages and disadvantages of ETV, TDF, and TAF, so as to better guide clinical practice.

## Author contributions

Mao-bing Chen and Hua Wang proposed the concept of this study and designed this systematic review. Mao-bing Chen registered the protocol of the systematic review and meta-analysis. Mao-bing Chen, Qi-han Zheng, Xu-wen Zheng and Hua Wang were responsible for the collection, collation and statistical processing of the literature. All authors participated in the drafting of the first draft of the paper. Mao-bing Chen reviewed and proofread the paper. All authors agree to publish the paper publicly.

**Conceptualization:** Mao-bing Chen, Hua Wang.

**Data curation:** Mao-bing Chen, Qi-han Zheng, Xu-wen Zheng, Hua Wang.

**Methodology:** Mao-bing Chen, Wei-yan Cui.

**Software:** Mao-bing Chen, Hua-lan Xu.

**Supervision:** Mao-bing Chen, Qi-han Zheng.

**Writing – original draft:** Mao-bing Chen, Hua Wang, Qi-han Zheng, Xu-wen Zheng, Hua-lan Xu, Wei-yan Cui.

**Writing – review & editing:** Mao-bing Chen.
